# Free Triiodothyronine and Cholesterol Levels in Euthyroid Elderly T2DM Patients

**DOI:** 10.1155/2012/420370

**Published:** 2012-09-03

**Authors:** F. Strollo, I. Carucci, M. Morè, G. Marico, G. Strollo, M. A. Masini, S. Gentile

**Affiliations:** ^1^Endocrine Unit, INRCA, Via Cassia 1167, 00189 Rome, Italy; ^2^Department of Clinical and Experimental Medicine, Second University of Naples, Via Pansini 5, 80131 Naples, Italy; ^3^Endocrinology and Diabetes Service, FBF St. Peter's Hospital, Via Cassia 600, 001879 Rome, Italy; ^4^DipTeRis, University of Genoa, Viale Benedetto XV 3, 16132 Genoa, Italy

## Abstract

Thyroid function regulates lipid metabolism. Despite the fact that T2DM is more prevalent in the elderly, often associates with thyroid dysfunction and increases cardiovascular risk both *per se* and via high TC and LDL-C levels, the association of the latter with FT_3_ and FT_4_ levels has not yet been fully investigated in T2DM. While trying to fill this gap in 296 elderly outpatients with T2DM, we found that TC and LDL-C correlated negatively with FT_4_ and positively with FT_3_. When divided according to treatment by oral hypoglycaemic agents (OHA) and insulin (IT), they reacted differently with respect to investigated associations: in the OHA's TC and LDL-C correlated negatively with FT_4_ and showed no association with FT_3_, whereas, in the IT's TC and LDL-C correlated positively with FT_3_ and negatively with FT_4_. When controlled for possible confounding factors, these associations did not change in the IT's but were missing in the OHA's. Recent literature reports upon complex hypothalamic and peripheral interactions between T2DM and thyroid, and suggests T_3_ to enhance cholesterol synthesis and to have a role in insulin resistance states. Further investigations are needed to understand the intimate mechanisms of lipid metabolism in T2DM with respect to thyroid function.

## 1. Introduction 

Thyroid function affects both lipid profiles and metabolic parameters [1]. Thyroxine (T_4_) and triiodothyronine (T_3_), the two main thyroid hormones, are secreted after thyroglobulin macropinocytosis and hydrolysis under the stimulation of pituitary thyrotropin (TSH) and are present in the blood mainly in noncovalent interactions with thyroxine-binding globulin, prealbumin, and albumin. Only 0.4% T_3_ and 0.04% T_4_ dynamically escape binding and, since free to interact directly with peripheral organs and tissues, are called free T_3_ (FT_3_) and free T_4_ (FT_4_) and are the only fractions believed to be metabolically active [2].

FT_4_ levels are about 100 times higher than those of FT_3_, which is considered to be the most active form of thyroid hormone and into which FT_4_ is converted within the peripheral tissue [3]. Inside a variety of cells, both interact with different affinity with *α*1, *α*2, *β*1, and *β*2 nuclear receptors in regulating the expression of target genes through the so-called thyroid response elements (TRE's), thus exerting the typical physiological functions which characterize them. These are mostly represented by enhanced calorigenesis, increased stroke volume and heart rate, enhanced sensitivity to catecholamines, bone turnover, gluconeogenesis, glycogenolysis, and lipolysis. The latter is mainly due to enhanced lipoprotein-lipase activity and increased hepatic low-density lipoprotein (LDL) receptor concentrations [4]. Besides genomic effects, widespread rapid onset nongenomic effects of FT_3_ and FT_4_ have been reported involving membrane-signalling pathways [5], the real extent of which has not been well defined yet [6].

When dealing with lipid metabolism, circulating T_4_ concentrations have always been found to be inversely associated with total (TC), high-density lipoprotein (HDL-C), and LDL cholesterol (LDL-C) levels [7]. In fact, high TC levels in hypothyroidism are caused by a reduction in LDL receptors [8] and L-T_4_ administration has a hypolipidemic effect in hypothyroidism, which is characterized by high thyrotropin (TSH) concentrations, meant at compensating for low FT_4_ levels. Thus, there is a greater benefit in patients displaying higher pretreatment TC or LDL-C and TSH despite the difficulty to identify any well-defined cut-off threshold for the association of the latter with lipids [9]. As a matter of fact, 4.3% hypercholesterolemic patients have been reported to be hypothyroid [10]. 

A contributing factor to high cholesterol levels in hypothyroidism is represented also by low FT_3_. Under normal conditions, the latter has the control over sterol regulatory element-binding protein-1 (SREBP-1), a crucial step for the expression of the LDL receptor, rather than over SREBP-2 [11]. However, it also acts directly by inducing at least two LDL-receptor TRE's in the liver [12]. Finally, increased flow of bile acids as a result of FT_3_ and FT_4_ is known to reduce cholesterol levels by depletion of its hepatic pool; however, such effect is counterbalanced by enhanced synthesis and uptake in the liver, eventually increasing TC and LDL-C under unfavourable circumstances [13].

Based on the considerations mentioned above, the relationship between thyroid function and the metabolic syndrome (MetS) has become a subject of interest for many research groups during the last few years [14], leading to the conclusion that even low-normal T_4_ levels may contribute to increased cardiovascular risk associated with lipoid abnormalities in people with the MetS [15]. Some of these studies reported that T_3_ behaved in a different way, being positively associated with body mass index (BMI) and waist girth [16–18]. A higher compensatory conversion of T_4_ to T_3_ to increase thermogenesis in obesity was the hypothesized mechanism [19, 20].

Diabetes mellitus (DM), in particular type 2 (T2DM), which is mostly associated with lipoid abnormalities and displays an increasing prevalence with age [21], is also known to dramatically increase cardiovascular risk (CVR) at any age [22]. Age does not seem to affect FT_3_ and FT_4_ levels in the human [23], whereas people with DM have been reported to suffer from thyroid dysfunction twice as much as the nondiabetic population [24]. Nevertheless, despite the fact that TC and LDL-C are major CVR factors identified as crucial targets in all current diabetes treatment guidelines [25], no extensive investigation can be found in the literature concerning the relation between thyroid and lipids in T2DM. This link is missing in the clinical management of people with DM now that carbohydrate response element-binding protein (ChREBP)—a newly identified lipogenic glucose-sensing transcription factor controlling hepatic lipogenesis—is known to be positively controlled by T_3_ in mammals [26] through its binding to thyroid receptor *β*-1. In fact, T_3_ has been shown to modulate hepatic lipogenesis through reciprocal regulation of SREBP-1c and ChREBP gene expression [27]. Moreover, T_3_ is endowed with a lipogenic effect via ChREBP enhancement in white adipose tissue, where both *α* and *β* thyroid receptors are expressed but only the *β* isoform is active with regard to that effect [28]. Thus, glucose, lipids, and thyroid hormones seem to interact according to a more complex mathematical function than as previously expected.

Taking into account the considerations mentioned above, with the present study, we evaluated the relations among FT_3_, FT_4_, TC, and LDL-C in elderly euthyroid people with T2DM.

## 2. Materials and Methods

We retrospectively evaluated clinical records from 350 outpatients, ages 70 years and older, referred to our clinic for T2DM during the last three years. All of them had thyroid function routinely evaluated in terms of FT_3_, FT_4_, and TSH concentrations. The study protocol was approved by the ethical committee. Exclusion criteria included smoking and any complications worse than background retinopathy, microalbuminuria, low-grade neuropathy, and nonobstructive arteriopathy. We also did not accept for this study patients with overt heart, liver or kidney failure, hypo/hyperthyroidism, or those on any medications possibly interfering with thyroid function. Thus, 296 people were qualified for study (245 women, 51 men), all taking statins since the time of diagnosis (4,5 ± 2,7 years) in order to prevent cardiovascular complications according to Italian Diabetes Guidelines [29]. They were either under oral hypoglycaemic agents (OHA subgroup, *n* = 196), invariably consisting of sulphonylureas and metformin, or under insulin treatment (IT subgroup, *n* = 100, of which 63 on 4 basal-bolus injections, the others with 2 or 3 injections as needed). Both subgroups followed a thorough self-monitoring blood glucose supervision associated with a strong empowering strategy [30].

Blood was drawn in our laboratory in the morning, after a 12-hour overnight fast. Chemistry was measured by Kodak Blood Multiple Analyzer and thyroid hormones by Immulite 2000 Immunoassay System.

Data evaluation was based upon SPSS 13.0 for descriptive (mean ± SD) and correlation analysis. Correlation analysis was performed first on all cases and then completed by partial correlation analysis applied to each subgroup (namely, OHA and IT), controlling for possible confounding factors. The least statistical significance of the differences among the means and of the associations was set at *P* < 0.05. 

For clarification concerning the abbreviations used within the text, please refer to the list at the end of the paper.

## 3. Results

The means and the standard deviations (S.D.) of all recorded clinical parameters are presented in Table 1.

When analysing all the cases, we observed a positive correlation of FT_3_ with both TC (*r* = 0.144, *P* < 0.05) and LDL-C (*r* = 0.161, *P* < 0.02). Conversely, FT_4_ correlated negatively with TC and LDL-C (*r* = −0.131, *P* < 0.05 and *r* = −0.134, *P* < 0.05, resp.). The disease duration and HbA1c were found to correlate negatively with LDL-C (*r* = −0.108, *P* < 0.05 and *r* = −0.144, *P* < 0.02, resp.). To avoid confounding effects, we introduced them together with other potentially interfering factors that are possibly related to thyroid function (age, BMI, and blood pressure) into partial correlation analysis of total and LDL-C with FT_3_ and FT_4_. As a result, no changes in correlation coefficient signs or significances were observed.

At this point, when we proceeded to further analyze the variance of observed parameters between OHA and IT subgroups, we found that the two were statistically homogenous with each other, as shown in Table 2. 

Though, in terms of correlation analysis, the two subgroups behaved differently in terms of thyroid hormones. In fact, in the OHA, total and LDL cholesterol showed no correlation with FT_3_ (*r* = 0.037 and *r* = 0.098, resp., nonsignificant) but correlated negatively with FT_4_ (*r* = −0.144, *P* < 0.05 and *r* = −0,145, *P* < 0,05). Conversely, in the IT, total, and LDL cholesterol correlated positively with FT_3_ (*r* = 0.291, *P* < 0.01 and *r* = 0.275, *P* < 0.02, resp.) while maintaining the same negative correlation with FT_4_, as observed in the OHA (*r* = −0.239, *P* < 0.05 and *r* = −0.186, *P* < 0.05). Moreover, the IT revealed a previously “hidden” negative correlation between HDL cholesterol and FT_4_ (*r* = −0.302, *P* < 0.01).

**Figure 1:**
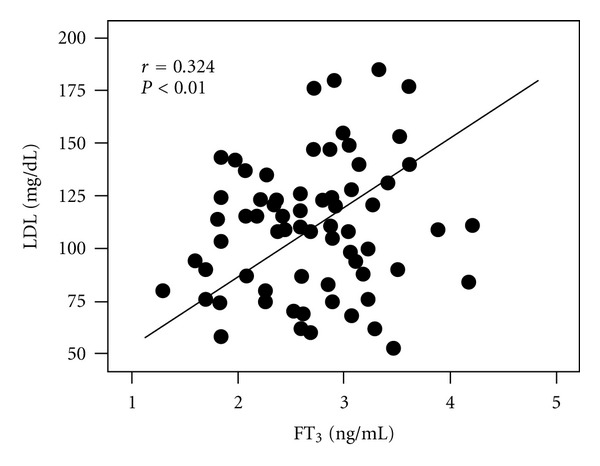
Association between FT_3_ and LDL cholesterol in IT-treated T2DM subjects controlling for age, BMI disease duration, blood pressure, and HbA1c.

We then repeated the same correlation analysis controlling lipid to thyroid relationship for age, BMI, HbA1c, blood pressure, and disease duration within each subgroup. As clearly shown in Figure 1, we could therefore confirm the positive correlation of FT_3_ with LDL cholesterol. Moreover, as summarized in Table 3, all the correlations previously described before were confirmed, including the one linking FT_4_ to HDL cholesterol. 

## 4. Discussion 

Before discussing our results, we will try to summarize them. TC and LDL-C displayed opposite correlations with FT_3_ (positive) and FT_4_ (negative) in elderly euthyroid subjects with T2DM. While a negative correlation would have been easy to accept and understand for thyroid hormones, a positive one was totally unexpected according to current concepts. When repeating the analysis in the two different subgroups, controlling them for confounding factors, we confirmed the above findings in the IT subgroup but not in the OHA subgroup. 

However, regarding the clinical implications of our results, a crucial point is that elderly people with T2DM carry the burden of a high CVR and have to be treated as carefully as their younger counterpart since morbility/mortality increases together with their LDL-C levels. With this in mind, diabetologists often concentrate on glucose, lipids, and blood pressure and generally do not take into account thyroid hormones in the absence of typical symptoms of thyroid dysfunction [31]. On the other hand, subclinical hypo- and hyperthyroidism are not rare at all. As geriatric endocrinologists, we prefer to assay thyroid hormones in diabetic elderly patients in order to rule out any subtle thyroid malfunction during the course of overall evaluation. 

Such a habit allowed us to collect data from a number of euthyroid elderly people with T2DM and to perform a correlation analysis between cholesterol and thyroid hormones. With regard to FT_4_, the analysis confirmed that thyroid function negatively correlates with TC and LDL-C, a trend commonly found in the general population. However, quite unexpectedly FT_3_ behaved the opposite way, being positively associated with TC and LDL-C. We then analyzed the association between thyroid hormones and all possible confounding factors. We found that age correlated positively and HbA1c and negatively to TC and LDL-C. Therefore, we introduced these two factors and other potentially interfering parameters including BMI, blood pressure, and disease duration as confounding factors, confirming their associations by performing partial correlation analysis.

After we divided our subjects into OHA and IT subgroups, the analysis of variance showed that they were fully homogeneous in terms of recorded clinical parameters. The only difference was in their treatment, namely, metformin/sulphonylureas versus insulin. This made us more confident in trying to reveal eventual differences occurring in relations between lipid profile and thyroid hormones. In fact, this would allow us to check whether different associations within subgroups are possibly linked to different treatment regimens.

In the OHA group, TC and LDL-C displayed no correlation with FT_3_ but correlated negatively with FT_4_ (*P* < 0.05), whereas in the IT group, TC and LDL-C correlated positively with FT_3_ (*P* < 0.02) and negatively with FT_4_ (*P* < 0.05). Once again we performed partial correlation analysis in the IT subgroup and found that the positive association of lipids with FT_3_ became even stronger while that with FT_4_ remained the same (see Table 3); however, both vanished in the OHA subgroup. 

Such findings might seem contradictory, but, in fact, they fit well with the role played by FT_3_ on SREBP-1 and SREBP-2 control, and consequently on the expression of LDL receptor, with its ability to indirectly enhance cholesterol synthesis and uptake by the liver. Moreover, in some previous studies an unexpected positive association between FT_3_ and lipids was mentioned without indepth explanation, and therefore remained underestimated. For instance, De Pergola et al. [32] found that FT_3_ correlated negatively with HDL-C levels (*P* < 0.001), and, in multiple correlation analysis, maintained an independent positive association with age (*P* < 0.001), waist girth (*P* < 0.05) and insulin levels (*P* < 0.001), a proxy for insulin resistance. It is worthwhile noting that in the study of De Pergola et al., FT_3_ was also associated with smoking habits. Our study took into account only nonsmokers, thus ruling out *a priori* a possible strong confounding factor. Others confirmed the previously mentioned association between FT_3_ and MetS components [15] suggesting insulin-resistance to be the link between the thyroid and lipids. Therefore, T_3_ may act as a strong independent metabolic signal in euthyroid insulin-resistant T2DM patients [33, 34].

Interestingly enough, according to recent reports, T_3_ added to diets containing peanut oil increased serum lipids in rats, sometimes even up to 20-fold [35], whereas T_3_ was found to increase cholesterol biosynthesis in the liver through the activation of *de novo* protein synthesis [36, 37]. Furthermore, T_3_, insulin, and their combination markedly stimulate cholesterol synthesis in cultured human skin fibroblasts [38], and in recent studies FT_3_ has been even shown to exert a beta-cell protective effect [39, 40]. All above considerations might explain the association we found between FT_3_ and TC and LDL-C in our IT patients and not in our OHA patients. In fact, it seems as if in the presence of insulin resistance T_3_ may not be able to act as fully as in the case of an unopposed insulin signal, such as when metabolically effective exogenous insulin levels are attained. Still another possible explanation for our findings result from an eventual compensatory increase in FT_3_. In fact, based upon widely accepted lipid metabolism regulation mechanisms by thyroid hormones, it might be hypothesized that in our patients cholesterol synthesis might have been enhanced by exogenous insulin and by peripheral T_3_ conversion from T_4_ [41, 42].

Another apparently anomalous finding, regarding the negative correlation of HDL-C with FT_4_ levels (*P* < 0.01) but not with FT_3_, deserves more discussion. Since early studies in the field, HDL-C was found to decrease both in hypo- and in hyperthyroidism, thus indicating that such association followed a U-shaped curve and could, therefore, only be analyzed within the physiological range of FT_4_ concentrations [43, 44]. In fact, slightly different behaviour between the two hormones has been reported in the literature, including a stronger enhancing effect of HDL-C on T_4_ target cell penetration in comparison to T_3_ target cell penetration [45]. A pro-HDL and anti-LDL effect of T_3_ was also described [46]. These observations have been often overlooked, despite their potential pharmacologic utilization in the metabolic syndrome.

We are aware of the intrinsic limits of the retrospective cross-sectional character of our study, which refers to subjects over 70 years of age with T2DM and, therefore, allows no definite conclusions with regard to younger people or in terms of cause-effect relationships. Nevertheless, due to the different association patterns found in IT patients as compared to OHA patients, we feel it is worthwhile to further investigate the topic. 

Specifically, reported data prompts some reappraisal regarding the relationship among some hidden aspects of cholesterol, insulin, and thyroid hormone metabolism, eventually fostering new controlled studies concerning the role of T_3_ in T2DM and the possible pharmacological interferences that different drugs may have on it.

Based on our data, it seems more prudent to treat elderly hypothyroid patients with T_4_, without any T_3_ integration if they are on insulin. This might prevent the risk of letting their LDL-C increase and thus of increasing statin dosage. We still do not fully understand the meaning of the association we found between FT_3_ and cholesterol in IT people with T2DM. In other terms, the positive association might be the expression of a compensatory hormone response to spontaneous LDL-C increase, as already hypothesized for patients with the MetS [15, 33, 34] and in line with the complex DM-thyroid interactions occurring via hypothalamic glucose sensing mechanisms [47] (as recently reviewed by Duntas et al. [48]).

In conclusion, in order to clarify the pathophysiological mechanisms underlying the observed results further experimental and clinical follow-up studies are needed. Should the observed associations be confirmed by future investigations, in older T2DM patients it might be useful to include FT_3_ once again in thyroid test panels which today are mostly limited to screening TSH and confirmation FT_4_.

## Figures and Tables

**Table 1:** Clinical parameters (mean ± SD) in the 296 patients under evaluation.

	Mean	SD
Age (years)	76.4	5.2
BMI (Kg/m^2^)	31.5	7.4
Dis-Dur (years)	4.47	2.69
HbA1c (%)	7.95	2.08
TC (mg/dL)	188.5	40.4
HDL-C (mg/dL)	47.6	14.3
LDL-C (mg/dL)	111.0	35.5
TG (mg/dL)	148.6	81.4
SBP (mmHg)	136.1	16.5
DBP (mmHg)	74.8	11.4
FT_3_ (pg/mL)	2.76	0.77
FT_4_ (pg/mL)	12.5	5.9
TSH (mU/L)	1.44	1.35

**Table 2:** Clinical parameters recorded in the IT and OHA patients: none of them differs significantly between subgroups.

	IT subgroup		OHA subgroup	
	Mean	SD	Mean	SD
Age (years)	76.2	4.9	76.5	5.2
BMI (Kg/m^2^)	30.9	6.4	31.9	7.9
Dis-Dur (years)	5.01	2.80	4.13	2.47
HbA1c (%)	8.0	2.5	7.9	2.1
TC (mg/dL)	184.6	41.6	191.0	40.3
HDL-C (mg/dL)	46.1	13.2	48.6	14.8
LDL-C (mg/dL)	107.5	35.1	113.2	36.2
TG (mg/dL)	150.2	67.5	144.1	74.4
SBP (mmHg)	137.1	16.6	135.7	16.6
DBP (mmHg)	73.8	12.4	75.4	11.1
FT_3_ (pg/mL)	2.73	0.94	2.80	0.65
FT_4_ (pg/mL)	11.9	2.1	12.8	7.1
TSH (mU/L)	1.45	1.40	1.37	1.29

**Table 3:** Correlation coefficients of cholesterol and its LDL and HDL fractions to FT_3_ and FT_4_ in T2DM elderly patients controlling for age, BMI, disease duration, and HbA1c.

Control variables	Thyroid hormone	Group	Statistics	Total cholesterol	LDL cholesterol	HDL cholesterol
Age, BMI, disease duration, blood pressure, and HbA1c	FT_3_	IT	*r*	0.346	0.324	0.147
			*p*	0.006	0.009	n.s.
		OHA	*r*	−0.014	0.043	−0.038
			*p*	n.s.	n.s.	n.s.
	FT_4_	IT	*r*	−0.295	−0.241	−0.310
			*p*	0.019	0.050	0.013
		OHA	*r*	−0.149	−0.142	−0.119
			*p*	n.s.	n.s.	n.s.

IT: insulin treated; OHA: treated by oral hypoglycaemic agents.

## Abbreviations

BMI: Body mass index
ChREBP: Carbohydrate response element-binding protein
DBP: Diastolic blood pressure
Dis-Dur: Disease duration
DM: Diabetes mellitus
FT_3_: Free triiodothyronine
FT_4_: Free thyroxine
HbA1c: Glycated haemoglobin
HDL: High density lipoprotein
HDL-C: HDL cholesterol
IT: Insulin treated
LDL: Low-density lipoprotein
LDL-C: LDL cholesterol
OHA: Oral hypoglycaemic agents
SBP: Systolic blood pressure
SREBP: Sterol regulatory element-binding protein
T2DM: Type 2 diabetes mellitus
T_3_: Triiodothyronine
T_4_: Thyroxine
TC: Total cholesterol
TG: Triglycerides
TRE: Thyroid response element
TSH: Thyroid stimulating hormone.
